# Necrotizing Cellulitis and Myositis in a Neutropenic Leukemic Patient

**DOI:** 10.7759/cureus.14941

**Published:** 2021-05-10

**Authors:** Akankcha Alok, John Greene

**Affiliations:** 1 Infectious Disease, Moffitt Cancer Center, Tampa, USA; 2 Medicine, Kasturba Medical College, Manipal, IND; 3 Internal Medicine, Moffitt Cancer Center, Tampa, USA

**Keywords:** pyoderma gangrenosum, immunosuppressed, sweet syndrome, leukemia cutis, ecthyma gangrenosum, infections, angioimmunoblastic lymphadenopathy with dysproteinemia

## Abstract

In this article, we review a case of necrotizing cellulitis and myositis in a neutropenic leukemic patient. He underwent a series of investigations to reach the diagnosis of pyoderma gangrenosum (PG). The lesion improved dramatically after pertinent identification and initiation of appropriate treatment. The management of PG is exceedingly challenging due to a lack of proper clinical criteria for detection and guidelines for treatment. PG must be considered as a differential in patients with enlarging, sterile, necrotic lesions, unresponsive to prolonged broad-spectrum antibiotics. Prompt recognition can prevent deeper infections and the formation of a chronic open wound causing cosmetic disfigurement along with other catastrophic complications.

## Introduction

Pyoderma gangrenosum (PG) is a rare inflammatory skin condition with variable etiology. It is often associated with underlying systemic diseases including hematologic malignancies and autoimmune conditions. Lesions classically begin as erythematous papules, pustules, or nodules that progress to painful deep necrotic ulcers with violaceous undermined borders. It is often misdiagnosed as soft tissue or necrotizing infection and can coincidentally improve with systemic antibiotics and wound care. Patients get exposed to multiple broad-spectrum antibiotics due to lack of diagnostic specificity, thus, leading to an increased probability of superimposed infections and antimicrobial resistance.

## Case presentation

**Figure 1:**
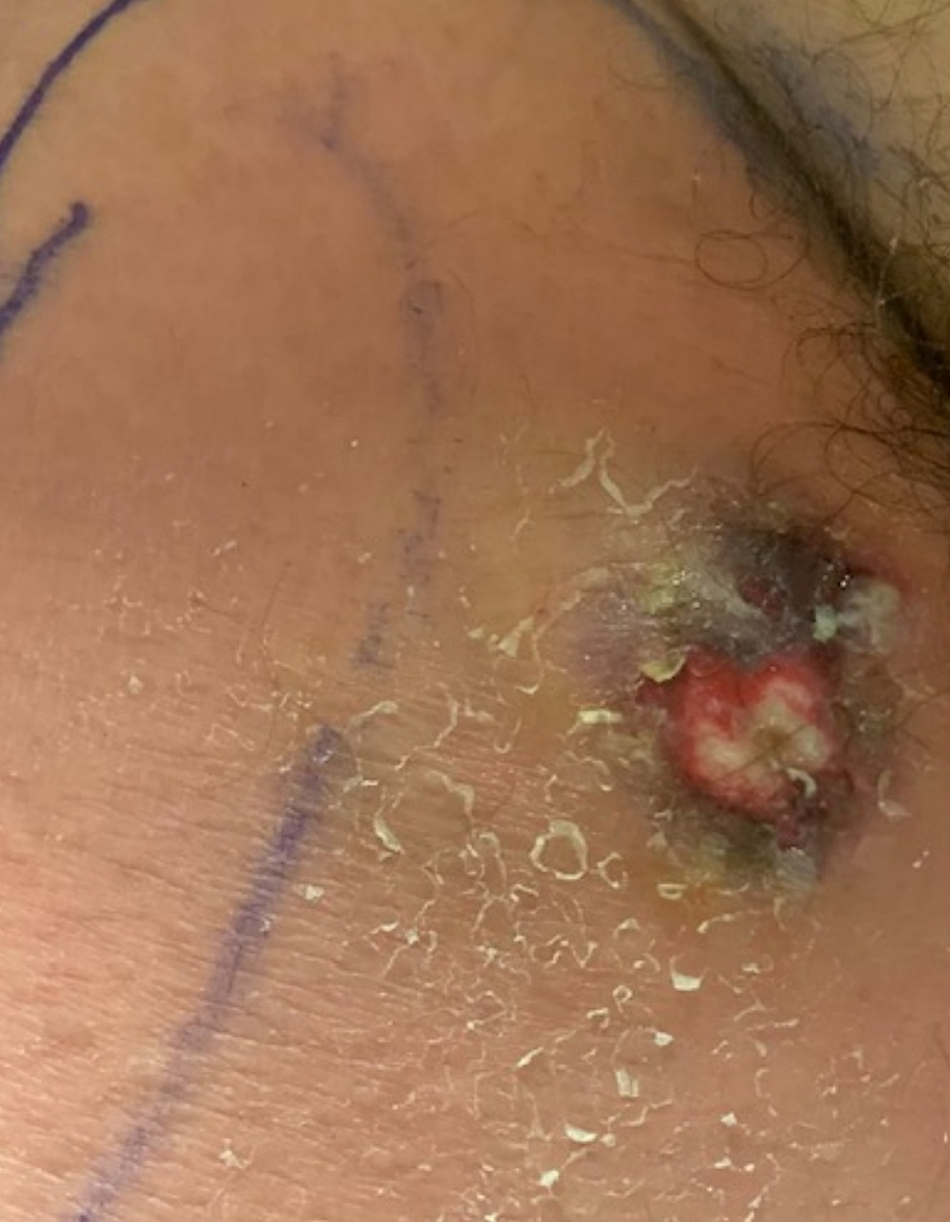
Appearance of the wound in the initial stages.

A 71-year-old Caucasian male with myelodysplastic syndrome presented with dull aching pain on his right medial thigh for the past two months, after finishing a course of chemotherapy. It was associated with difficulty in walking and intermittent high temperatures. He denied any trauma to the area. The patient did not indulge in alcohol, tobacco, or recreational drug use, and had no recent travel history. He was not on any medication at the time of presentation. On examination, his vital signs were blood pressure (BP) of 110/66 mmHg, respiratory rate (RR) of 12/minute, temperature of 102.2°F, and weight of 80 kilograms. Regional evaluation displayed an area of erythema and induration on the right medial proximal thigh that was warm, edematous, and tender to touch (Figure 1).

**Figure 2:**
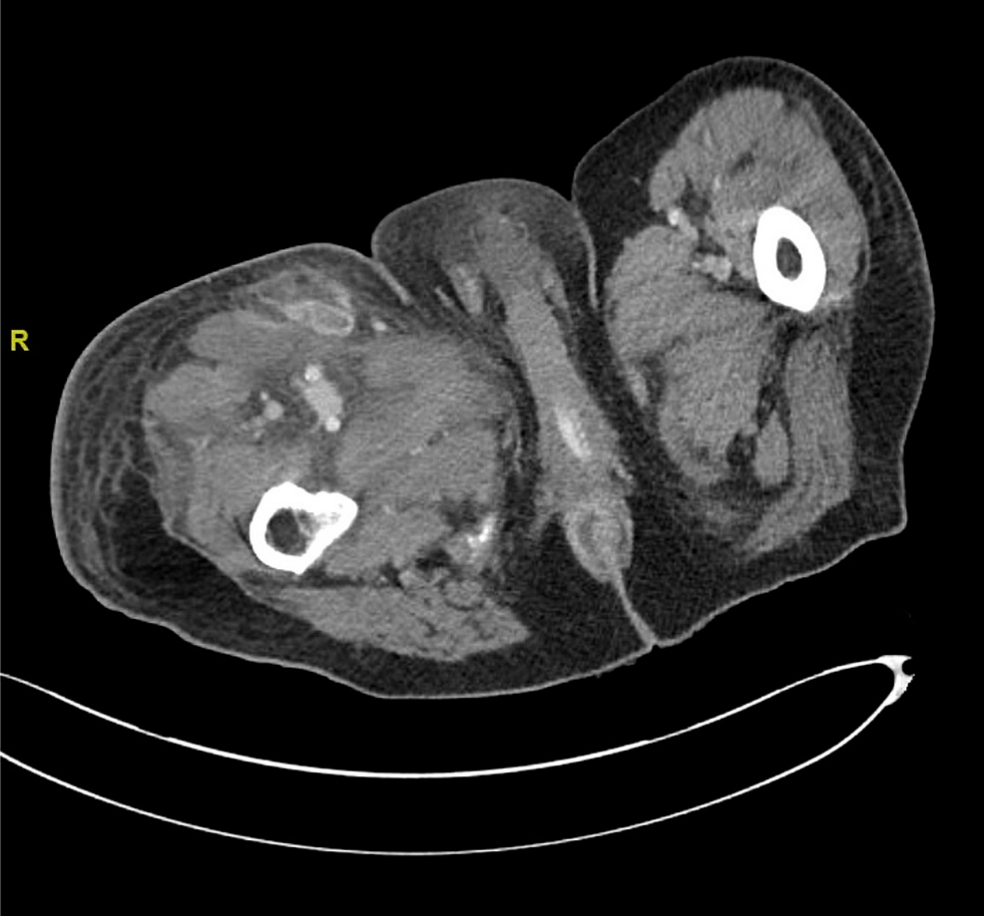
Findings on the first scan of the wound.

Physical examination was otherwise unremarkable. Significant laboratory findings included a white blood cell count (WBC) of 0.76 k/ul, absolute neutrophil count (ANC) of 0.26 k/ul, hemoglobin of 6 g/dl, and platelet count of 6 k/ul. The ANC had remained less than 0.5 k/ul for several months. Other laboratory findings were within the normal range. Soft tissue imaging of the area showed extensive subcutaneous and intramuscular edema but the absence of purulent inflammation (Figure 2).

**Figure 3:**
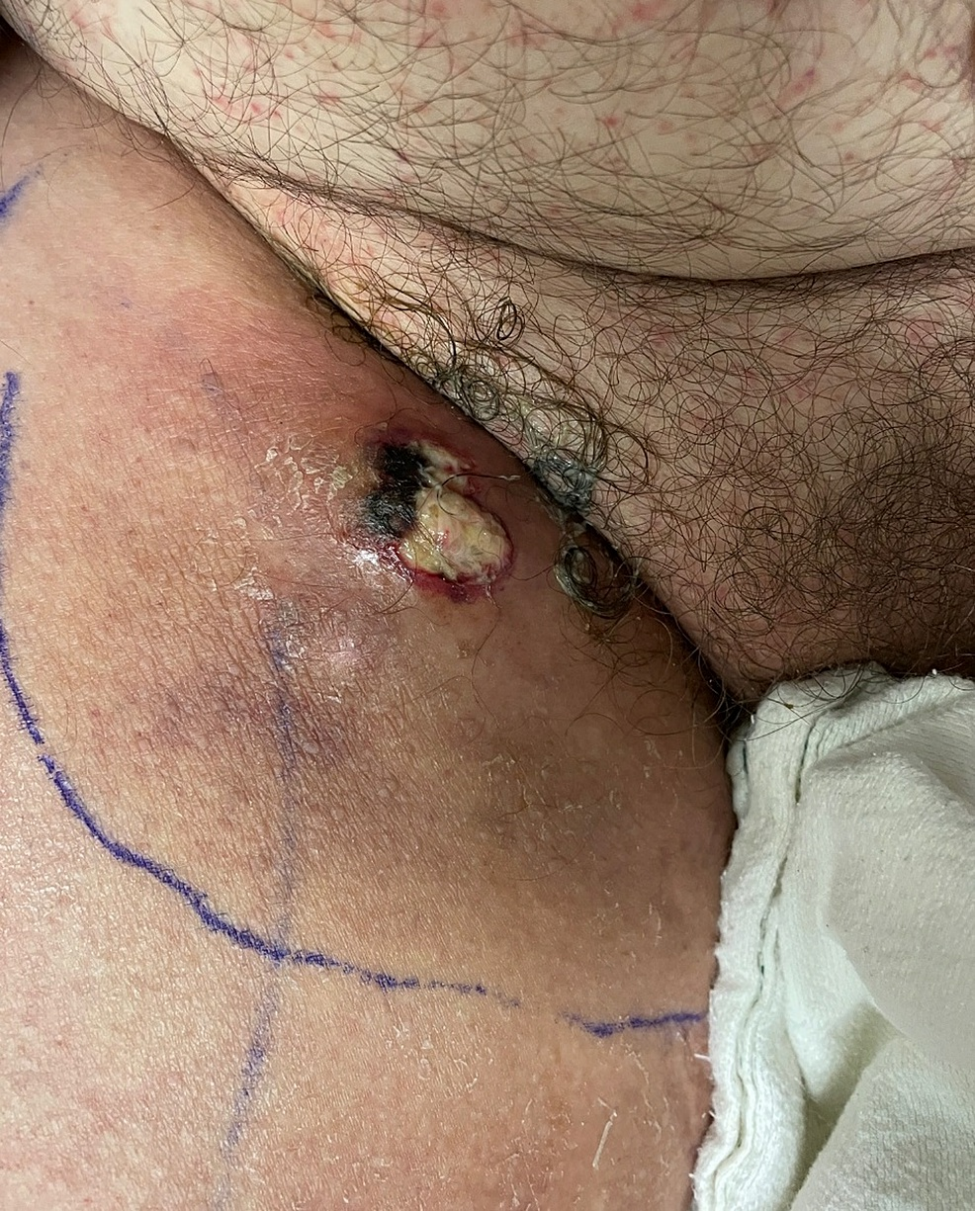
Progression of the wound with eschar.

A provisional diagnosis of cellulitis with deep tissue myositis was made and empiric treatment with cefepime and vancomycin was initiated. Extensive microbiological evaluation including blood, urine, superficial, and deep wound cultures was negative. Workup for viral, fungal, or acid-fast organisms was negative. Gross pathological examination of a small punch biopsy from the area was consistent with chronic inflammatory skin changes with an underlying fluid collection. The lesion evolved despite escalation to broad-spectrum antibiotics including clindamycin, meropenem, daptomycin, metronidazole, and tobramycin along with micafungin and liposomal amphotericin B for fungal coverage. It gradually developed into a friable, necrotic nodule with overlying eschar and vesicles (Figure 3).

**Figure 4:**
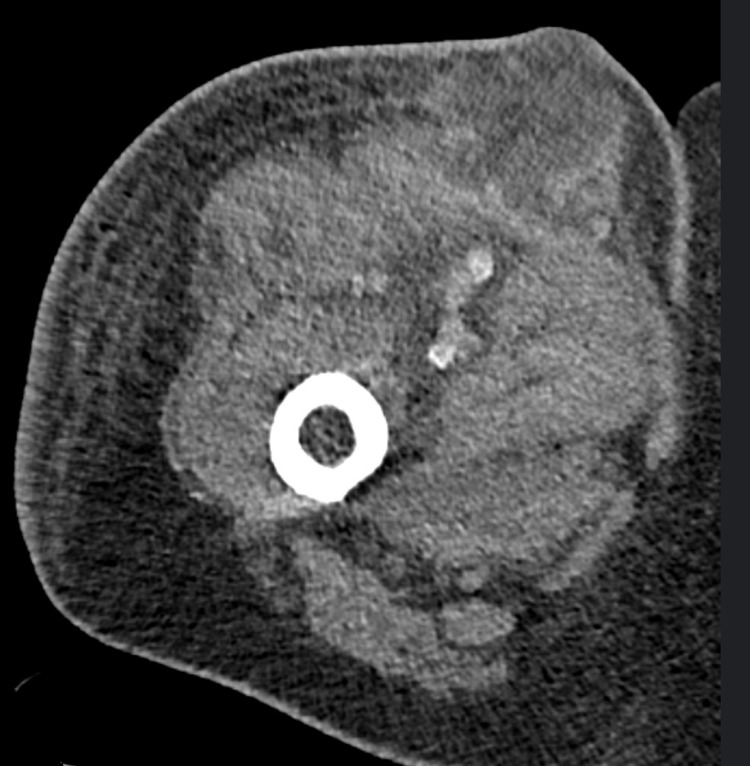
Subsequent scan showing growth of the lesion.

A repeat scan of the pelvis showed a subcutaneous mass-like density with peripheral enhancement, measuring 5.5 cm x 5.4 cm indicating a growing phlegmon (Figure 4).

**Figure 5:**
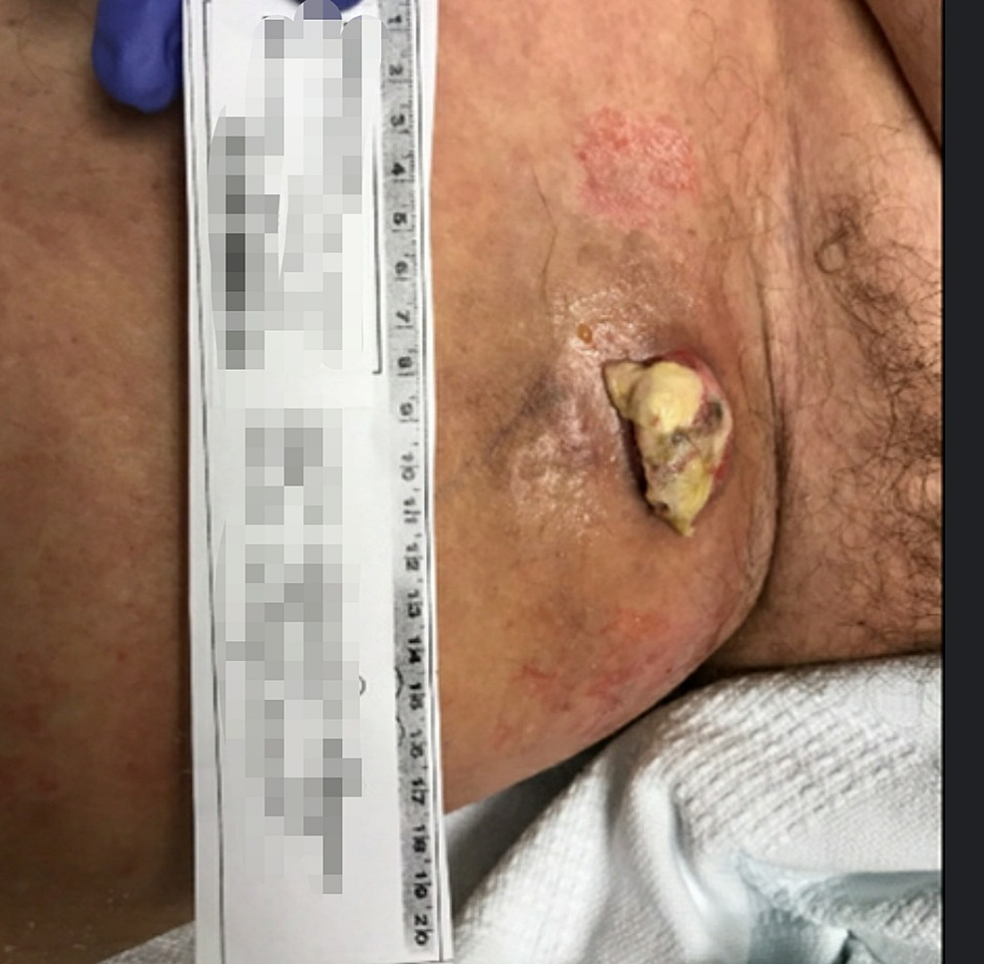
Full-blown phlegmon before biopsy.

**Figure 6:**
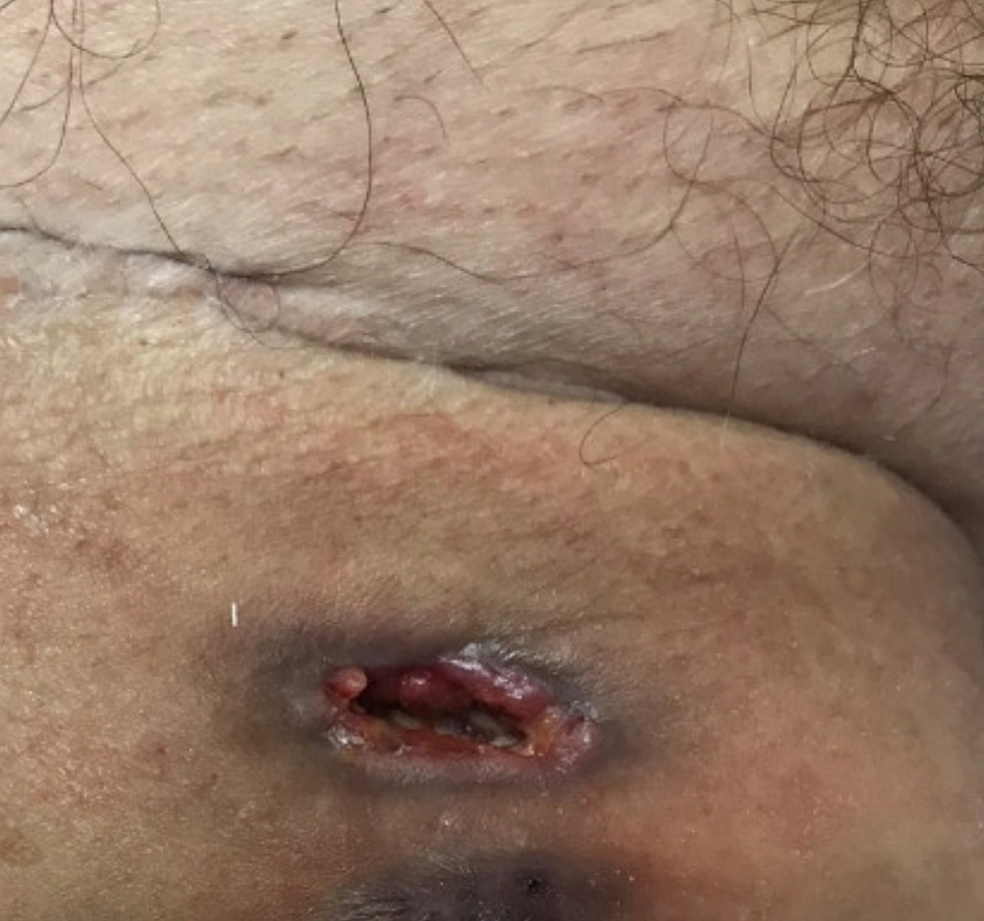
Open defect at wound site after biopsy.

The patient appeared symptomatically better after treatment with granulocyte colony-stimulating factor (GCSF) and was discharged on ciprofloxacin and doxycycline pending histopathological diagnosis. On follow-up admission, a serial scan depicted a localized open defect with previous fluid collection on the right thigh. A deeper wound biopsy reported fragments of necrotic tissue with reactive neutrophilic to infiltrate (Figures 5, 6).

A trial of systemic and intralesional steroids based on the pathology report demonstrated drastic improvement confirming the diagnosis of pyoderma gangrenosum. The patient was discharged on doxycycline, ciprofloxacin, and oral prednisolone which was gradually tapered, with no recurrence of symptoms.

## Discussion

This case served as an ideal example of the variable fashion in which PG can be interpreted. It also highlighted the diagnostic dilemmas while treating a necrotizing phlegmon. Initially, a deeper biopsy was not feasible due to thrombocytopenia. The surgical team recommended not cutting into the lesion due to a high risk of infection but drainage was warranted given the interval development of the fluid collection. The wound eventually started looking like a phlegmon and resecting it in the setting of neutropenia could form a large nonhealing defect. Finally, only histopathology could confirm the diagnosis of PG and saved further apprehension. A trial of intralesional corticosteroids was avoided before confirmation of the diagnosis to prevent the flaring up of possible infections.

 Pyoderma gangrenosum is predominantly a diagnosis of exclusion as indicated in this case report (Table 1) [1-3].

**Table 1:** Systemic diseases associated with pyoderma gangrenosum. PAPA: pyogenic arthritis, pyoderma gangrenosum, and acne; PAPASH: pyogenic arthritis, pyoderma gangrenosum, acne, and hidradenitis suppurativa; SAPHO: synovitis, acne, pustulosis, hyperostosis, and osteitis; TNF-alpha: tumor necrosis factor-alpha; COPD: chronic obstructive pulmonary disease

Gastrointestinal disorders	Hematological disorders	Autoimmune disorders	Inherited autoinflammatory disorders	Other associations
Ulcerative colitis	Leukemia	Rheumatoid arthritis	PAPA	Thyroid diseases
Crohn’s disease	Multiple myeloma and its variants	Seronegative arthritis	PAPASH	Solid-organ malignancies
Autoimmune hepatitis	Myelodysplastic syndrome	Systemic lupus erythematosus	SAPHO	Melanomas
Hepatitis C	Polycythemia vera			Sarcoidosis
Primary biliary cirrhosis	Lymphoma			COPD
				Vasculitis
				Congenital immunodeficiencies
				HIV
				Drugs like TNF-alpha inhibitors

Pathologically, it is classified as a neutrophilic dermatosis which established the final diagnosis in our patient. PG has an unpredictable clinical course as portrayed in our report. Patients can frequently complain of malaise and arthralgia along with the lesion. It also exhibits pathergy, which is a phenomenon of exaggerated skin injury and delayed healing after minor trauma. Surgical intervention should therefore be avoided to prevent rapid progression of the disease before a definite diagnosis is obtained. Besides corticosteroids, immunosuppressive medications like cyclosporine can also be used to treat PG but have limited applicability when it comes to already immunocompromised patients like ours and are, therefore, reserved for resistant cases. It is important to discuss the possible differential diagnoses of the disease to distinguish it better (Table 2) and to understand the rare incidence of PG in hematological malignancies (Table 3) [4-6].

**Table 2:** Differential diagnosis of pyoderma gangrenosum.

Infectious	Non-infectious
Bacterial:	Cutaneous lymphoma
Ecthyma gangrenosum	Sweet syndrome
Cellulitis	Vasculitis
Impetigo	Antiphospholipid syndrome
Atypical mycobacteria	Panniculitis
Cutaneous tuberculosis	Arterial ulcers
Syphilitic gumma	Venous insufficiency and ulceration
Cutaneous anthrax	Necrobiosis lipoidica
Viral:	Arthropod bite reaction
Herpes simplex	Iododerma
Cytomegalovirus	Bromoderma
Fungal:	Factitious ulcer
Cutaneous mucor	Skin cancers
Blastomycosis	Drugs:
Sporotrichosis	Hydroxyurea
Coccidioidomycosis	Granulocyte stimulating factor
Parasitic:	
Cutaneous leishmaniasis	
Cutaneous trypanosomiasis	

**Table 3:** Incidence of pyoderma gangrenosum in hematological malignancies. PG: pyoderma gangrenosum; MDS: myelodysplastic syndrome; AML: acute myeloid leukemia; MGUS: monoclonal gammopathy of undetermined significance; CML: chronic myeloid leukemia

Malignancy	Number of PG cases reported in the literature
MDS	83
MGUS	75
AML	39
Multiple myeloma	22
CML	19
Polycythemia vera	18
Primary myelofibrosis	15
B-cell non-Hodgkin lymphoma	10
Chronic myelomonocytic leukemia	8
Chronic lymphocytic leukemia	8
Hairy cell leukemia	7
T cell lymphoma	7
Cutaneous T cell lymphoma	7
Hodgkin lymphoma	4
Follicular lymphoma	3
Essential thrombocythemia	3
Waldenstrom macroglobulinemia	3
Chronic neutrophilic leukemia	1
Chronic eosinophilic leukemia	1
Myeloproliferative disease, unspecified	1
Mixed phenotype acute leukemia	1
T lymphoblastic leukemia/lymphoma	1
Plasmablastic lymphoma	1
Mantle cell lymphoma	1
Non-Hodgkin lymphoma: diffuse, mixed cellular type	1
Non-Hodgkin lymphoma, unspecified	1

Cellulitis was the initial differential of interest, due to the commencing erythematous and tender lesion coexisting with fever. However, negative bacterial cultures and evolution of the wound into an open non-healing ulcerated defect excluded cellulitis. The patient also developed transient vesicles on the wound, but a negative herpes simplex virus (HSV) and cytomegalovirus (CMV) polymerase chain reaction (PCR) ruled out possible viral etiology. A non-healing ulcer in a neutropenic environment can often hint at tubercular or fungal origin, but a negative acid-fast and silver stain combined with negative cultures made such a situation improbable [7].

Sweet syndrome is an acute neutrophilic dermatosis exhibiting fever, rash and erythematous, non-ulcerated plaques and nodules often accompanied by leukocytosis and neutrophilia. It occurs due to cytokine-mediated hypersensitivity and can be associated with inflammatory disorders and hematological malignancies. Treatment includes the administration of oral prednisolone. The biopsy showed neutrophilic dermatosis in our case, but the lack of generalized non-ulcerated rash and absence of specific lab findings made Sweet syndrome less likely [8].

Leukemia cutis (LC) is the infiltration of the skin with neoplastic leukocytes. It can present as hyperpigmented nodules and plaques in the setting of hematological malignancies. The lesions can develop into blisters, erosions, and ulcers with surrounding petechiae and purpura. This condition can be managed with systemic chemotherapy targeting the underlying malignancy. Local therapy may include electron beam radiation and phototherapy. The absence of malignant cells in the histopathology of the wound rules out LC in our patient [9,10].

Ecthyma gangrenosum (EG) is a bacterial infection most commonly caused by Pseudomonas aeruginosa primarily in neutropenic patients. It presents as a painless gangrenous ulcer with a central area of necrosis. Tissue biopsy and culture lead to the ultimate diagnosis. Although other anti-pseudomonal antibiotics are also known to be effective, a combination of beta-lactam antibiotics and aminoglycosides is considered suitable for treatment in high suspicion of multi-drug resistance. Surgical debridement is sought in recalcitrant cases. The absence of tissue-associated gram-negative bacteria and positive blood cultures along with lack of response to appropriate antibiotic therapy ruled out EG in the case reported above [11].

 Angioimmunoblastic lymphadenopathy with dysproteinemia (AILD) is confirmed by histological examination of a lymph node under direct immunofluorescence showing vasculitis, perivascular C3, and granular IgM accumulation. It is characterized by hypergammaglobulinemia and increased IgE levels, none of which could be appreciated in our case. Since AILD is a lymphoproliferative disorder presenting with fever and occurring with other malignancies, it is considered to be a differential diagnosis for PG with neutropenia [12].

## Conclusions

Leukemic patients are more likely to have Sweet syndrome and leukemia cutis especially when monocytic leukemias predominate. EG is more common in the setting of prolonged profound neutropenia and is usually located in the region of the thigh and perineum, as exhibited by our patient. PG, therefore, is quite rare in the setting of refractory hematological malignancy and leads to an increased hospital stay and aggressive antimicrobial therapy before it is diagnosed with a deep skin biopsy and treated with corticosteroid therapy. However, after establishing the diagnosis and beginning relevant treatment, PG generally has a good prognosis, unless it is complicated by extensive surgical procedures or unrestricted antibiotic use leading to super-infections. Deeper ulcers can heal with scarring and the recurrence rate is variable at the same or different body sites. Overall, a multidisciplinary and holistic approach involving dermatologists, oncologists, infectious disease specialists, and surgeons, is quintessential. An amalgamation of timely identification, accurate topical care, and addressing the underlying cause is key to the management of pyoderma gangrenosum.

## References

[1] Ha JW, Hahm JF, Kim KS (2018). A case of pyoderma gangrenosum with myelodysplastic syndrome. Ann Dermatol.

[2] Saffie MF, Shroff A (2018). A case of pyoderma gangrenosum misdiagnosed as necrotizing infection: a potential diagnostic catastrophe. Case Rep Infect Dis.

[3] Shahid S, Myszor M, De Silva A (2014). Pyoderma gangrenosum as a first presentation of inflammatory bowel disease. BMJ case reports.

[4] Montagnon CM, Fracica EA, Patel AA (2020). Pyoderma gangrenosum in hematologic malignancies: a systematic review. J Am Acad Dermatol.

[5] Carøe C, Fogh K (2019). Pyoderma gangrenosum associated with melanoma. Wounds Middle East 2015.

[6] Long H, Su Y, Lu Q (2014). Rapidly progressing leg ulcer with fever in a woman with chronic diarrhea. JAMA.

[7] To D, Wong A, Montessori V (2014). Atypical pyoderma gangrenosum mimicking an infectious process. Case Rep Infect Dis.

[8] Maller B, Bigness A, Moino D, Greene J (2020). Sweet’s syndrome associated with hematological malignancies. Leuk Res.

[9] Zang PD, Adler BL, Hughes M, Harter N, DeClerck B, Luu M (2019). Leukemia cutis: an unusual localization in the groin mimicking infection. Lancet Oncol.

[10] Rao AG, Danturty I (2012). Leukemia cutis. Indian J Dermatol.

[11] Khwaja SI, Yacoub AT, Katayama M, Greene J (2015). Ecthyma gangrenosum: a multidrug-resistant gram-negative bacilli—a growing fear in the immunocompromised patients. Infect Dis Clin Pract.

[12] Böni R, Dummer R, Dommann-Scherrer C, Dommann S, Zimmermann DR, Joller-Jemelka H, Burg G (1995). Necrotizing herpes zoster mimicking relapse of vasculitis in angioimmunoblastic lymphadenopathy with dysproteinaemia. Br J Dermatol.

